# Music performance as knowledge acquisition: a review and preliminary conceptual framework

**DOI:** 10.3389/fpsyg.2024.1331806

**Published:** 2024-02-07

**Authors:** Mark Reybrouck, Andrea Schiavio

**Affiliations:** ^1^Musicology Research Unit, KU Leuven, Leuven, Belgium; ^2^Department of Musicology, IPEM, Ghent University, Ghent, Belgium; ^3^School of Arts and Creative Technologies, University of York, York, United Kingdom

**Keywords:** embodied cognition, motor control, sensorimotor integration, music performance, skill acquisition

## Abstract

To what extent does playing a musical instrument contribute to an individual’s construction of knowledge? This paper aims to address this question by examining music performance from an embodied perspective and offering a narrative-style review of the main literature on the topic. Drawing from both older theoretical frameworks on motor learning and more recent theories on sensorimotor coupling and integration, this paper seeks to challenge and juxtapose established ideas with contemporary views inspired by recent work on embodied cognitive science. By doing so we advocate a *centripetal* approach to music performance, contrasting the prevalent *centrifugal* perspective: the sounds produced during performance not only originate from bodily action (centrifugal), but also cyclically return to it (centripetal). This perspective suggests that playing music involves a dynamic integration of both external and internal factors, transcending mere output-oriented actions and revealing music performance as a form of knowledge acquisition based on real-time sensorimotor experience.

## 1 Introduction

Cutting across the boundaries of well-defined areas of research such as motor learning, skill acquisition, cognition, and expressive behavior, music performance can be conceived of as a distinct field of study—one which arguably finds its place within the broader realms of perception and action studies—with a special focus on expert behavior (Lehmann and Davidson, 2002; Alexander, 2003; Chaffin et al., 2003; Ericsson et al., 2007; Brown et al., 2015), creativity (Deliège and Wiggins, 2006; Loui, 2018; Schiavio et al., 2022c), embodiment (Leman, 2007; Cox, 2016; Reybrouck, 2021), interaction (Lesaffre et al., 2017; Reybrouck, 2023a) and dynamic system theory (Richardson and Chemero, 2014; Nijs et al., 2023). Due to this interdisciplinary nature, a number of contributions have approached the study of music performance through comparisons with other domains, such as sports (see Schiavio et al., 2019 for an overview) as well as seemingly distant areas including mathematics and chess (Ericsson et al., 2007). This comparative approach is enlightening as it brings together descriptive and explanatory methodologies from a range of distinct disciplines. By bridging domains that may appear to lean toward the more intellectual aspects of cognition (e.g., chess) and those also heavily reliant on motor components (e.g., sports), these contributions emphasize the close relationships between perception, action, and cognition that we also find at the heart of music performance.

The close integration of perception, action, and cognition lies also at the core of an approach known as embodied cognitive science. This is an umbrella term describing an interdisciplinary school of thought that examines mind and subjectivity as phenomena arising from the profound connection between low-level (i.e., sensorimotor) and high-level (intellectual) processes (see Berthoz, 1997; O’Regan and Noë, 2001; Noë, 2004). This framework advocates the view that mentality is co-determined by body and brain, and that it cannot be reduced to brain activity or computational processes (see Maturana and Varela, 1986; Johnson, 1987, 2007; Varela et al., 1991; Lakoff and Johnson, 1999; Thompson and Varela, 2001; Borghi, 2005; Leman, 2007; Cox, 2016; Reybrouck, 2021; van der Schyff et al., 2022 for musical applications). Among others, compelling evidence in support of this approach emerges from studies that illustrate how cognitive systems actively seek and acquire information to enable controlled interactions with their environments. These studies delve into the coadaptation of the central nervous system and the physical constraints of the human body during motor learning, encompassing both ontogenetic and phylogenetic levels (see Wolpert et al., 2001; Davidson and Wolpert, 2003; Davids et al., 2008; Tani et al., 2008; Di Paolo, 2010; Clark, 2011). The embodied approach has ushered in a paradigm shift within cognitive science and music cognition research. Nevertheless, the field as a whole is still in a state of development and is characterized by the usual challenges of newly emerging disciplines: it grapples with numerous divergent and sometimes contradictory assertions, requiring operational definitions and the enhancement of conceptual clarity in the employed terminology. Music performance serves as an ideal domain through which we can attain conceptual clarity for embodied cognitive science, offering many real-life cases that could provide evidence of the deep interaction of perception, action and cognition. Simultaneously, this endeavor has the potential to contribute a new theoretical framework adept at integrating conceptual insights and empirical findings into a coherent whole, thereby fostering a richer understanding of what music performance truly entails in experiential and sensorimotor terms. This article aims at partially filling this need, focusing on this latter objective.

Our primary goal is therefore to examine music performance through a theoretical lens that embraces the embodied perspectives just outlined. In this exploration we juxtapose two perspectives: a *centrifugal* viewpoint and a *centripetal* one: we posit that music performance needs not just be understood as an outcome-driven behavior that originates from bodily action (i.e., the centrifugal perspective), but also as a pathway for acquiring knowledge grounded in the immediate experience of the music being played (i.e., the centripetal perspective) and the actions underpinning it. This may also encompass visual elements related to controlling movements, interactions with other musicians, and the interpretation of notated music. In light of this, it can be argued that music performers engage in a process of knowledge acquisition that draws from various information sources: the acoustic properties of the sound produced, the tactile and kinesthetic sensations linked to bodily movements for sound creation, visual cues from musical notation (in the case of score reading), and the visual monitoring of movements, among other factors. The explanatory power of the move sheds light on the intricate nature of musical performance cognition, offering novel insights that may help us better understand the web of embodied interactions through which music-making and musical experience emerge.^1^

In what follows, we first offer a review of a range of theoretical frameworks and empirical findings to provide a coherent picture of the existing background literature. This synthesis aims to offer a cohesive overview of current and previous studies. Subsequently, we advocate for an expansion of explanatory frameworks by situating music performance studies within the broader context of embodied cognition and general knowledge construction.^2^ Following this section, we turn our attention to our central question and ask: what type of knowledge does music performance generate? To answer to this question we should navigate the literature from a novel viewpoint and acknowledge the complexity of the phenomenon under scrutiny to examine how the fundamental structural elements of musical performance can be perceived as a cohesive whole during the act of making music. Among other things, our analysis will also consider two key concepts: *motor* var*iability* and *practice*. The former, in particular, will receive special attention, given its pivotal role in the initial phase of learning and, in turn, the construction of specific knowledge.

### 1.1 A preliminary example

**Figure 1:**
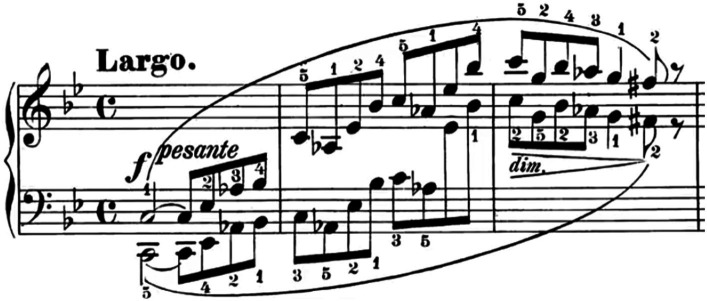
First three bars of Chopin’s first ballade Op. 23.

Consider an amateur pianist who is motivated to listen to technically challenging piano pieces such as Etudes by Chopin or Rachmaninov in preparation to their own performance. Even if the music is “understood” in a music-analytical sense, the transition from mere listening to sight-reading increases the level of engagement with sounds. The efforts to decipher the score and translate the notes into motor patterns confront the pianist with a lot of sensory information (aural, visual, tactile, kinesthetic, proprioceptive) which is not available in the case of mere listening. The learning process that characterizes the efforts to master a performance can be viewed as an ongoing process of updating, correcting, and adding new routines. All of these activities contribute to the construction of procedural knowledge, which can also be activated at a later moment, particularly in a listening-without-playing context (as described below). This picture shows the tension between “knowledge that” and “knowledge how,” which manifests itself in the actual performance (see Dreyfus, 2002). As reported by Pavese (2022), the disparity between these two types of knowledge was thoroughly examined by Ryle (1949). He presented classic challenges to the widely-held intellectualist view, which asserts that “knowledge how” is essentially equivalent to “knowledge that.” Ryle proposed an anti-intellectualist perspective, contending that “knowledge how” and “knowledge that” are separate forms of knowledge. But while analytically separate, there is a mutual interaction between the two. Figure 1 provides an example. It depicts the first three measures of Chopin’s first ballade. Decoding the score is not particularly challenging since both the left and right hands execute the same notes at octave intervals. The difficulty does not also lie in memorizing the note sequence, which collectively creates a coherent musical expression. Rather, the true challenge emerges in the finger placement while playing. Even when the pianist precisely knows what to play, the fingers may seem to operate independently of cognitive control, requiring considerable effort to render these three bars fluid. Yet, through persevering in this challenging process, the pianist confronts the motoric elements of performance, merging them with the auditory feedback from the produced sounds and the visual cues from the score or observing the fingers.

This sets the stage for questions about motor performance as a constitutive element of musical skills. A distinction should be made, however, between the motor “learning stage” and the stage of “accomplished skill acquisition.” The exploratory character of the former has an additional value with respect to knowledge construction and adaptive behavior, and it can be argued that a kind of regression to this early stage of motor learning should be preserved also in later skilled performance, somewhat analogous to Werner’s genetic principle of “spirality” in the broader context of his *orthogenetic principle of development* (Werner and Kaplan, 1963; Werner, 2013, see also Bibace and Kharlamov, 2013; Glick, 2013; Müller et al., 2013). At the core of this concept is the idea that throughout ontogenetic development lower-level processes and functions do not disappear but resurface—hence the term “spiraling”—again under specific conditions, both pathological and non-pathological. It is tempting, therefore, to argue for a kind of dynamic coexistence of schematizing processes and crystallized end-forms of acquired skills on the one hand, and tentative early exploratory behavior on the other hand, rather than claiming a unidirectional trajectory of development that proceeds from a diffuse and syncretic to a more articulate stage, in which seemingly unrelated elements are integrated in a more coherent way (see Høffding and Schiavio, 2019; Schiavio and Kimmel, 2021). It is a promising approach that may function as a kind of glue between divergent approaches to motor learning and control.

## 2 Theoretical frameworks and paradigms: an overview

As anticipated, music performance can be studied from several perspectives. There is, first, the broader field of skill acquisition and expert behavior, with major contributions from domains outside of the realm of music. Secondly, there is the approach that starts from *motor learning and control* in general, with an ongoing debate between adherents of “central” and “peripheral” theories There are, thirdly, the contributions from cognitive science and philosophy of mind, with a particular focus on the *embodied approach* to cognition. And there is, finally, the *experiential approach to cognition*, which revives, as it were, the older insights of pragmatic philosophy. It is not the aim of this contribution to provide a detailed review of each of them following this structure. Instead, we elaborate rather selectively on some of their commonalities with the aim to present a more coherent picture of how music performance can be understood as a process of knowledge acquisition grounded in real-time, sensorimotor experience.

### 2.1 Skill acquisition and expert behavior in general

Skill acquisition research has a long history. Being oriented initially at what might be seen as high-level forms of cognition, such as playing chess or learning languages, more recent research has been directed also at lower-level, visceral aspects of mental life, including the study of motor learning (Davidson and Wolpert, 2003). Several theoretical approaches have tried to uncover the underlying mechanisms of skill acquisition, such as association theories, neuromaturation theories, stage theories of motor learning, information processing theories, and neurocomputational theories. Some of them are quite general, others are more narrowly restricted to specific domains of expertise: information processing theories conceive of the brain as a computational device that processes sensory information with the help of stored representations of the world (Adams, 1971; Schmidt, 1975); neurocomputational theories explain how representations allow learners to create mental models of the world (Davids et al., 2001); Fitts’ motor stage theory distinguishes between a cognitive phase (what to do) with effortful, tentative, slow and inaccurate performance, an associative or motor stage with slow learning and performance adjustments and implicit mechanisms taking over, and an autonomous stage where the skill becomes largely automatic, with little deployment of attentional resources (Fitts, 1964). The studies on the achievement of motor skills and musical performance, in particular, have contributed considerably to the understanding of processes such as the acquisition of expertise, attention, and automaticity of movement coordination and control, with a shift toward a more balanced emphasis on cognition, perception, and action (see Davids et al., 2008 for an overview).

Nevertheless, numerous unresolved issues and conflicting statements persist, hindering the formation of a cohesive understanding of the broader field. Particularly notable in the literature are significant divides such as the distinction between central and peripheral theories of motor learning, the debate over the role of internalized schemes or motor programs vs. the ongoing control of movement based on continuous feedback, and the differentiation between general-purpose abilities and more specific skills confined to the narrow domain of music performance. While these perspectives have frequently been portrayed in a conflicting and dichotomous manner, they may also be seen as complementary facets of the same coin. Hence, we have the opportunity to integrate these distinct approaches within a more comprehensive framework, wherein the embodied cognition approach plays a pivotal role. With this in mind, we can compare older theories of motor learning with more recent ones focusing on sensorimotor coupling and integration, which are directly inspired by the embodied approach. This comparison illustrates how past insights can ignite new avenues for theorizing and empirical research. And this seems to be quite urgent, as there are currently several areas of research working independently and without knowledge of each other. Table 1 provides a preliminary overview that brings together some major concepts of existing previous and current theories and conceptualizations.

**Table 1:** Schematic, non-exhaustive overview of some major theories and concepts related to skill acquisition, expert behavior, motor learning and sensorimotor integration (including music-specific ones).

Theories/concepts	References	Major findings/claims
**Skill acquisition**		
Sill acquisition in general	Miller (1956), Rosenbaum et al. (2001), and Gobet and Lane (2012)	Skill acquisition progresses from processing and executing component task units at the bottom level to achieving Gestalt processing at the top level. This involves the grouping of information into meaningful chunks.
	Cundey (1978), Ericsson et al. (1993), Rosenbaum et al. (2001), and Gladwell (2008)	Skill is a capacity to achieve a goal within a specific domain enhanced through practice. It manifests itself as proficient, swift, and accurate performance, encompassing a broad spectrum of mental activities.
Cognitive and perceptual-motor skills	Newell and Rosenbloom (1981)	Effect of practice time on performance for cognitive and perceptual-motor skills.
Association theories	Woodworth (1899), Thorndike (1927), and Skinner (1938)	Relationship between movement stimuli and action; major focus on observable performance.
Neuromaturation theories	Gesell (1928)	Motor development is the outcome of growth and maturation; motor learning is the outcome of practice and experience.
Information processing theories	Adams (1971) and Schmidt (1975)	Brain as computational device that governs behavior; processing of information guides skillful activity.
Neurocomputational theories	Willingham (1998), Kawato (1999), Davids et al. (2001), and Wolpert et al. (2001)	Mental representations construct mental models of the world; identification of brain regions that control action; coping with multiple information streams; communication between sensory representations and motor system; control-based learning; forward and inverse modeling.
Skills and motor behavior	Adams (1971)	Skills encompass a wide domain of possible behaviors; skills must be learned and are defined by motor performance in attainment of task-specific goal.
**Expert behavior**		
Expert behavior	Lehmann and Davidson (2002), Alexander (2003), Chaffin et al. (2003), Ericsson et al. (2007), and Brown et al. (2015)	Expertise is maximal adaptation of performer to task-environment.
Expert performance	Hurley (2002)	Carrying out actions or processes that are intentional but not consciously intended.
	Jacobs and Michaels (2006)	Expert performance as part of dynamic system comprising performer, tools, environment and other performers or individuals.
**Motor learning and control**		
Motor stages theory	Fitts (1964)	Three stages of learning: cognitive phase (effortful, tentative, slow, inaccurate); associative or motor phase (slow, adjustments, more implicit); autonomous stage (automatic, little attentional resources).
Central theories of motor learning	Henry and Rogers (1960) and Keele (1968)	Representation of perceptual-motor information within CNS; storage of motor commands; motor programs; open loop control.
Peripheral theories of motor learning	Adams (1971)	Use of ongoing performance-related feedback to control movement; closed-loop theory
Schema theory of discrete learning	Schmidt (1975)	Combines open- and closed-loop control; set of rules (schema) about execution of movements linked to received feedback during performance; variable practice conditions facilitate schema creation.
Perceptual representation of action effect	Elsner and Hommel (2001), Van Orden et al. (2003), Davids et al. (2008)	Actions are represented by codes of their anticipated effect; integration of movement patterns and perceptual effects; integration and automatic storage; construction of internal models of the world and movements; role of CNS in modeling of behavior.
**Sensorimotor integration and embodied cognition**		
Sensorimotor coupling	Berthoz (1997), O’Regan and Noë (2001), Noë (2004), and Borghi (2005)	Mental life is co-determined by body and brain.
	Drost et al. (2005), Lahav et al. (2007), Shelton and Kumar (2010), Chen et al. (2012), Brown et al. (2015), and Thaut et al. (2015)	Auditory-motor coupling; sound-action association.
Intersensory translation	Hubbard and Stoeckig (1992), Gordon (1997), Halpern (2001), Aleman and Wout (2004), and Brodsky et al. (2008)	Visual-motor coupling; sight-reading; audiation; kinesthetic-like covert phonatory processes; notational audiation.
	Drost et al. (2005)	Intersensory and sensorimotor translation processes.
Embodied approach	Johnson (1987, 2007), Maturana (1987), Varela et al. (1991), Lakoff and Johnson (1999), Thompson and Varela (2001), Stewart et al. (2010), and Hutto and Myin (2013).	Integration of perception, action and cognition; living organism as brain–body system geared for action and interaction with environment.
Embodiment in music	Leman (2007), Cox (2016), Reybrouck (2021), and van der Schyff et al. (2022)	In all its manifestations, music can be seen as a phenomenon rooted in action.

### 2.2 Musical expert behavior

The performance by a virtuoso music performer is an interesting starting point to begin our exploration. It demonstrates one of the most demanding cognitive and motor achievements of human beings, facing challenges such as speed, dexterity, and precision. In addition to these challenges, individuals may also play from memory and incorporate expressive nuances into the performance (Brown et al., 2015). As such, music-making combines motor, auditory, high-level cognitive, and expressive processes during performance. Together, these processes constitute five basic types of musical abilities: sight-reading, performing rehearsed repertoire, playing from memory, playing by ear, and improvising (McPherson, 1995b; McPherson and Thompson, 1998). It can be questioned, however, whether these basic types are orthogonal categories in a statistical sense, or whether they rely on more basic dimensions, which, together, add up to build a more generic conception of musical expert behavior. We are inclined to favor the latter view by distinguishing between the actual production of sound and those processes that prepare or modulate its execution, either in real time or outside of the time of performing. It can be asked, moreover, what sets musical expert behavior apart from everyday movements and motor skills in general. Two characteristics seem to be critical in this regard: *speed* and *precision*. Expert pianists can hit more than 20 notes per second with less than 3% errors and with millisecond precision in timing, which is in stark contrast to everyday movements which tend to be much slower when the need for precision increases (see Brown et al., 2015 for an overview). Both characteristics can be subsumed under the umbrella term of *sensorimotor integration*, with several integration loops—such as the audio-motor, the visual-motor, the tactile-motor, and even the proprioceptive-motor coupling—which couple action to perception. There is the primary feedback from manual or manual-podalic—as in the case of a drummer or organ player who also use the feet—motor processing, besides feedback from kinesthetic phonatory motor processing and phonatory resources during subvocalization as measured by covert vocal fold activity during silently reading music notation (Brodsky et al., 2008). The question can be raised, however, to what extent this feedback become part of conscious experience (see below).

Positing such broader systems offers the advantage of transcending distinct performance categories, such as playing from memory or sight-reading. It opens up new perspectives for a *multisensory approach* to music performance by stressing the importance of the real-time experience of produced sounds (Reybrouck and Eerola, 2017; Reybrouck, 2019). Not all experiences, however, are perceptually-bound, somewhat related to the ontological status of the temporal unfolding of the sounding music. There are, in fact, three different time moments that modulate the actual experience of the music: before, during, and after. These moments can be separated conceptually in terms of anticipation, current experience, and reflection afterwards, but they overlap and interconnect also to some extent. It is a conception that echoes the older theories of time put forward by Husserl and Schütz (see Reybrouck, 2004 for an overview). Husserl’s phenomenological analysis of time-consciousness has been pioneering in this regard (see Gallagher, 2017 for an overview). Elaborating on the constitution of time, he has sketched a phenomenological description of the temporality of experience, stating that he basic unit of time consciousness is not a strict knife-edge present, but a thick duration block or temporal field that contains the three temporal modes of past, present, and future. He accordingly introduced the concepts of “temporal window” or actual “now-moment” and the twin concepts of “retention” (what has just passed) and “protention” (what is to come next) in perception and experience (Husserl, 1928). Schütz, on the other hand, recalling Bergson’s “tensions of consciousness” and James’ “stream of consciousness,” conceived of inner life as a stream of connected experiences, with an ontological distinction between five dimensions of inner time: reproduction, retention, now, protention, and anticipation. The “now-moment” is the time of immediate experience, the past is a set of complete but indirect experiences which are available through memory, and the future is available through anticipation. There is, however, an ontological difference between the constituent parts of past and future: retention refers to the experience of an immediate past in which the actual experience is still retained; reproduction refers to the past but is not directly contiguous with the actual experience; protention refers to the immediate future; and anticipation is directed to a more distant future (Schütz, 1971, 1976).

These older insights sound amazingly modern, especially in the context of theories of motor learning. They provide some clues to resolve apparent contradictions between so-called “central” and “peripheral” motor theories by giving a more balanced weight to memory, experience, and anticipation, and by also giving some new impetus to the much-challenged conception of *mental representation*. Very generally speaking, current theories of expertise often tend to assume that expert performance results from the increased mental storage of exemplars, chunks (Barsalou, 2003). It is a conception that calls forth the role of internal models which are based on (mental representations of) desired performative outcomes, and which offer an excellent scaffolding through the learning trajectory (Lehmann, 1997; Gruhn, 2006; Hallam and Bautista, 2018). They are mainly built up through hours of study and practicing, being shaped by self-monitoring and evaluation strategies (McPhail, 2013; Lehmann and Jørgensen, 2018).

Such mental models have been described also in the domain of motor skill acquisition in the sense that internal processes give rise to relatively permanent changes in the learner’s movement capabilities (Schmidt and Wrisberg, 2004). Riding a bicycle, catching a ball, or driving a car are examples of skills that require substantial practice for effective performance. Once mastered, however, these skills enable more efficient interaction with the environment. They facilitate the detection of important and useful information, allowing for timely responses through the generation of coordination patterns that adapt more easily to varying performance characteristics (Davids et al., 2006). The significance of the adaptive nature of such skills challenges the dependence on conscious processing and explicit rule-following, which is conventionally assumed to result in tacit knowledge. Instead, it advocates for a more fluid and flexible understanding of how optimal motor skills are developed (Bril, 2002). Much is to be expected here from neuroscientific studies, which have shown that plastic adaptations occur as the result of training with changes in both the structure and function of the somatosensory/motor system. Such plastic changes have been found in the auditory cortex, the motor and premotor regions with their white-matter pathways, and the corpus callosum of trained musicians (see Brown et al., 2015 for an overview).

A decisive factor in defining the level of skill acquisition or expertise, further, is the *automaticity* and/or *efficiency* of the adaptive motor mechanisms. It is generally assumed that there is a progressive shift from an initial phase in which skills are acquired to the final stage of performance where tasks can be executed without any cognitive involvement. The role of consciousness is quite important here, with heated debates about the respective roles of the conscious as against the unconscious mode in motor learning (Willingham, 1998). The former generates target end points for movements together with their sequence; the latter relies on perceptual-motor integration. Both modes can be available through training, but the conscious one is more accurate, be it at the expense of greater attentional cost. The conscious mode is most prevalent at the beginning of the acquisition process (Papineau, 2013), but also at those moments where the performer’s absorption fades away or is not totally ready. Conscious conceptualizing and explicit reflections, therefore, typically occur before skill acquisition or after a skillful performance, either as a temporary scaffold to automatize routines or as a conceptual expedient to rationalize the routine a-posteriori (Cappuccio, 2015). The availability of two distinct modes of processing—i.e., the dual mode principle—has been corroborated also by cognitive and neuroscience studies, which have shown a distinction between unconscious neural representations and their (optional) conscious counterparts. Indeed, research on preconscious processing clearly indicates that conscious states may not accompany unconscious processing, and if they do, they mostly follow rather than preceding it. Conscious awareness, moreover, falls away also as automaticity develops during skill acquisition, with unconscious mechanisms taking over control. It seems, therefore, that different neural mechanisms underlie conscious and unconscious processing (see Barsalou, 1999 for an overview).

Another issue in the context of skill acquisition is the persistent debate about musical talent as predisposition or talent as against experience and practice (Ericsson et al., 1993; Krampe and Ericsson, 1996; Sloboda et al., 1996). There are the famous examples of child prodigies like Mozart and others, as well as retrospective studies on the development of expertise with the wide-spread notion that expert performers began their training before the age of six (Manturzewska, 1990; Lehmann, 1997; Jørgensen, 2001), suggesting that there might be a sensitive period for musical training (Knudsen, 2004; Bailey and Penhune, 2012) with some evidence from morphometric studies who showed structural differences—gray- and white-matter structures—in the corpus callosum and the sensorimotor cortex (Schlaug, 1995; Amunts et al., 1997; Steele et al., 2013). It has been stated also that professional level performance requires more than 10,000 h or a minimum of 10 years of targeted practice (Ericsson et al., 1993; Gladwell, 2008). This dispositional approach and the actual level of execution—the performance outcomes—however, are less relevant from a learning perspective, which aims at preparing for and improving actual performance practice. Skill acquisition, in fact, is a gradual process that occurs over many performance attempts. It is typically understood by examining the dynamics of performance changes over time (Davids et al., 2008).

More interesting, therefore, is to uncover how expert executors manage to automatize their performance during extensive rehearsals in preparation for performance in addition to building and maintaining their skills. It is a fruitful domain of study, which requires longitudinal case studies of expert performers who prepare for performance, thus providing a window into the cognitive processes that are involved in the development of high-level skill. The behavioral reports of practicing musicians provide a glimpse of how expert performers have a detailed understanding of why they start, stop, back up, and repeat during practice, and how they constantly make decisions about technique, interpretation, and performance (Hallam, 1995a,b; Chaffin and Imreh, 2001). Several strategies have been described in this regard, with different stages of proceeding in which performers focus on different features of the acquisition stage: an initial focus on the musical structure, a stage that engages in technical aspects of performance and interpretation, and a final stage that is directed at the expressive qualities to be conveyed (Chaffin and Logan, 2006; Chaffin et al., 2009).

It should be mentioned, finally, that music performance cannot be reduced merely to motor learning and sensorimotor skills. Equally important are *mental phenomena* such as memory and imagination (which, according to the embodied approach, are continuous with the former ones). Playing from memory, in particular, is a major challenge for professional soloists, with extraordinary demands. It makes sense, therefore, to rely on theories that are developed to account for *skilled memory* in other domains, such as chess and acting (Ericsson and Kintsch, 1995), intuitive problem solving (Gobet and Simon, 1996) and motor skills (Van Orden et al., 2003) and to apply them to the realm of music. It may be argued, in this regard, that skilled music performers memorize mainly in the same way, with only minor differences that relate to the music, the instrument, and their particular learning style. Three general principles seem to characterize the features their expert memorizing: meaningful encoding of new information in terms of stored schemas (such as chords, scales, arpeggios, etc.) which are the result of training; the use of a firmly established retrieval scheme that provides access to the chunks of information in long-term memory (such as the formal structure of the music); and prolonged and extended retrieval practice that allows them to decrease retrieval time to keep pace with the performance (Ericsson and Kintsch, 1995; Lehmann and Gruber, 2006; Chaffin et al., 2009). A distinction should be made, moreover, between domains that rely almost entirely on explicit memory and those of performing arts, where it is possible to rely also on automatic, implicit motor skills instead of explicit memorization. Musicians, in particular, tend to supplement their implicit memories of motor sequences with explicit conceptual memories, which act as a mental map to monitor and modify highly practiced motor sequences (Chaffin and Logan, 2006).

The coordination between encoding and executive functions is most prominently demonstrated in sight-reading. This intricate process entails simultaneously deciphering new material and executing it without relying on automated performance developed through deliberate practice. It is complex as it requires the processing of visual information—both reading and understanding—the motor control to translate this into movement patterns, and the processing of auditory information which acts also as a controller for adjustment of the performance in real time (Oswald et al., 2007; Herrero and Carriedo, 2019; Herrero and Carriedo, 2022). Significant predictors of high-level sight-reading are psychomotor speed, early acquired expertise, mental speed, and the ability for mental imagery, and to some extent also working memory (Kopiez and Lee, 2008).

### 2.3 Perception, action, and interaction

Playing a musical instrument is a multifaceted skill. It aims at the production of sounds by manipulating the instrument through specific movements which directly produce sensory feedback, thus linking sensory and motor processes. It can be asked, however, to what extent this can contribute to the performers’ understanding of the music (and of themselves) while playing. The term *performative awareness* of the body has been used in this regard. It is a kind of consciousness which allows performers to better recognize their feelings, movements, thoughts, and beliefs as being their own by stressing in particular the role of proprioception and revaluing the tactile and haptic dimension of musical engagement (Gallagher, 2005; Peñalba-Acitores, 2011).

Music performance, in this view, is not merely a motor phenomenon. It calls forth listening as well in a way that it may entrain motor facilitation. One could call this “enactive listening” (Reybrouck, 2021) in the sense that listeners may be able to re-enact those motor actions that are needed to perform the music as it is heard (Gordon et al., 2018). It is a way of listening that allows listeners—also performers are listeners—to perceive music through motor engagement (Schiavio et al., 2017), either manifest—by executing actual movements—or in imagination. The latter, however, is not necessarily perceptually-bound (Reybrouck, 2001; Prinz and Chater, 2005). It emphasizes an approach that aligns with those motor theories of perception that focus on the dynamic tension between “sensorimotor processing” and “ideomotor simulation.” The former aims at linking the incoming sensory input to the central nervous system and the effector organs in a continuous and ongoing way, with the aim to minimize possible deviations and keep disturbances within critical limits—hence the term *conservative behavior*; the latter takes more distance to the sensory input and allows the perceiver to simulate the actual unfolding of the stimuli at a virtual level of imagery (Paillard, 1990, 1994; Berthoz, 1996, 1997; Reybrouck, 2020b). Both approaches may complement each other in the sense that music processing affects not only the executive and the sensory systems; it may also activate mechanisms of mental simulations, entrainment, creativity, and social connectedness even in solitary practices (see Schiavio et al., 2015, 2022a,b,c, 2024). Taken together, however, they provide a richer and more holistic model of music processing that encompasses the whole body and its interactions with its environment. The notion of *interaction* is quite important here (see Reybrouck, 2023a for a broad overview). It should be seen as a dynamic approach to musical engagement that goes beyond a linear and unidirectional input–output model of information processing. The latter has for a long time been considered as a linear sequence, over cognitive planning, and representation of goals to a decision to act, and symbolic representations. A more modern view should try to overturn this so-called “sandwich model,” with cognition being sandwiched between the layers of sensory input and motor output, which are not considered to be cognitive themselves (Hurley, 2002). One of the proposed alternative approaches is the embodied approach we have introduced earlier, which conceives of living organisms as brain–body systems geared for action and interaction with their environments (see Stewart et al., 2010).

Rather than thinking in terms of causality, with input generating output, one can assume a kind of symmetric relationship between input and output, with input being able to modify the output and vice versa. It is more fruitful, therefore, to think in terms of *circularity* and *closed loops* rather than in terms of *open loops* that drive the motor output without relying on sensory feedback. Circularity basically means that the output is fed back to the input, allowing the performer to evaluate and control the output through flexible coordination of perception and action. The idea is implemented most typically in the example of a servomechanism that tries to keep disturbances within critical limits (i.e., conservative behavior, see above). It has figured as the central metaphor of cybernetics, involving a cyclic image of the brain and its environment, with internal sets of feedback loops themselves having feedback connections to the environment and being completed through it (McCulloch, 1989; Cariani, 2001). The closed loop metaphor has implications for motor learning, for perceptual learning, and also for musical sense-making in general. Motor learning, which aims at improving performance, is typical for organisms that interact with their environment by taking in sensory input and producing motor output. This can be done in a rigid way as exemplified by lower organisms that show no motor learning at all. In higher organism, however, there is a continuous need for learning, which increases when their environment, their body, and the tasks to be performed may change with development. Learning, in that case, is the only mechanism that is fast enough to master new tasks such as running on complex terrains, manipulating novel tools, writing, or dancing (Wolpert et al., 2001).

There are several strategies to learn to interact with the environment, either in a supervised, a reinforced, or unsupervised way. The supervised way is the easiest to assess as the environment provides an explicitly desired output or target for each input, which makes it possible to measure the performance of the learning system by the discrepancy between its output and the desired target. The unsupervised way, on the contrary, is more challenging as the environment provides inputs without giving desired targets or any measures for reward or punishment (Wolpert et al., 2001, p. 487). The approach is interesting as it opens up possibilities for exploratory behavior, which can be developed in autonomy or under supervision, both in-the-moment and in retrospect. The latter, in particular, has been studied already in the context of sports by using “re-enactment” techniques—such as, e.g., showing videos of the performance—to provide post-performance feedback to help athletes to reconnect the feelings and outcomes of their activities (Hauw, 2018; Gesbert et al., 2022). Strongest effects are obtained by integrating videos with a form of “reflective practice” so as to generate a re-enactment, as a kind of re-living of the experience itself, which, then, can be linked to a normative example (Hauw, 2009). Re-enactments techniques, however, are not limited to visual feedback. They may involve also auditory signals (Sors et al., 2015; Pizzera et al., 2017; Schaffert et al., 2019), and, in the case of music, they should even contain tactile and haptic information. The use of re-enactment techniques as reflective practice can be seen as a mediating tool, echoing the older insights by Vygotsky, who distinguished between tools that are “externally oriented” and serving as conductors of human influence on the object of activity, and those that are “internally oriented” with the aim to master oneself (Vygotsky, 1978). The concept of mediation was central in his writing, with a special emphasis on the use of *tools*, especially psychological tools, or signs. This means that our acting in the social and physical world does not proceed in a direct, unmediated way, but in an indirect way or mediated by signs (see Wertsch, 1981/1930 for a broader discussion).

This brings us to the use of *concepts* and *propositional symbols* in motor and perceptual learning. The symbolic approach has been strongly criticized in particular by theorists inspired by the conceptual tools of embodied cognition, who argue against the “disembodied” and “detached” nature of such a framework. They claim, on the contrary, that our perceived world is constituted through complex patterns of sensorimotor activity and that organisms “enact” or “bring forth” their worlds (Varela et al., 1991, p. 164; Thompson, 2007). Applied to music, it can be stated that the symbolic-conceptual approach can be criticized for its lack of connection to the sensory richness and temporal fine-tuning of the actual sounding music, as well as the downplay of the living body of the musician or the perceiver (see Schiavio and van der Schyff, 2016). As such, there has been a kind of paradigm shift in some fields of musicology to stress the role of the actual and lived experience in a real-time listening situation (Reybrouck, 2016, 2019, 2020a). This is the “experiential approach” (Lakoff, 1987, p. 120), which has been anticipated already in the field of cognitive linguistics, revolving around the concepts of embodied cognition and conceptual or non-objectivist semantics (see also Johnson, 1987). It has received some theoretical grounding in philosophical writings, in the field of psychology of perception in general, as well as empirical support from recent contributions from neurosciences and the neurobiology of music perception and performance. Yet the “conceptual-symbolic” approach has also its merits and benefits. It allows for plasticity and reversibility of mental operations, which is so typical of symbolic play. Being to some extent disconnected from the actual sensations, it is possible to rely on mental replicas rather than on the sounds themselves and to mentally navigate through these recollections in memory or anticipation, with less limitations and perceptual constraints (Reybrouck, 2006, 2016).

## 3 Music performance and the role of circularity

Research on musical performance is of recent date with the bulk of studies revolving around the capacity to re-create pre-existing compositions from notation. Furthermore, there remain numerous unresolved questions regarding the evaluation of performances that involve memory, playing by ear, and improvisation, as opposed to sight-reading and the execution of rehearsed music (McPherson, 1995a). From a cognitive and exploratory standpoint, the initial phases of “learning” in musical performance are particularly intriguing. These phases center around the intricate interplay of physical and mental processes with the actual sounds or their symbolic representations found in the musical score.

### 3.1 Motor learning and control

A major question with regard to motor learning and control is the *innate* or *acquired character* of our motor repertoire. Motor learning, in fact, is a process that evolves both during the individual lifetime and over generations, with a brain that adapts to control the body via the motor system. There are, as such, innate patterns of behavior which are driven by evolutionary forces with hardwired motor skills in the brain that are established even before birth. Such “innate wiring” may speed up motor skill acquisition and provide a starting point for future motor learning. It requires pre-specification of neural connections, which must be robust to possible perturbations, but at the expense of flexibility for learning novel skills. Motor learning, on the contrary, requires the breaking down of these rather rigid innate synergies as manifested in reflexes and central patterns generators (Wolpert et al., 2001). It is of a different nature than largely innate learning skills such as locomotion, chewing, and vestibulo-ocular responses, which are qualitatively different from other learned skills (Willingham, 1998). A distinction must be made, therefore, between *motor development* with behavioral changes that are the outcome of growth and maturation and *motor learning* where practice or experience is the determining factor (Haywood and Getchell, 2005). The former has been the subject of neuromaturational theories, with as major finding that maturation of the central nervous system acts as a catalyst for the development of new movement skills with a gradual disappearance of older, less functional movements (Davids et al., 2008).

Another major issue in motor learning is the theorizing about so-called *central* and *peripheral theories* of motor learning, together with accumulated neuroscientific evidence of the role of central nervous system functioning and psychological modeling of behavior. The central theories state that skilled performers store motor commands as abstract or generalizable rules to be applied in a variety of contexts, with parameters such as speed and force being adjusted before the execution and without external feedback (Keele, 1968; Schmidt, 1975; Schmidt and Lee, 2005; Magill, 2006). Musicians, in fact, can execute actions more quickly than the central nervous system can perceive them, which points in the direction of fast and fluent actions being produced by feedforward mechanisms that rely on motor commands that are already available, and which are selected and prepared before execution (Ruiz et al., 2009, and Brown et al., 2015 for an overview). The central theories, however, have been criticized by approaches that challenge internal, brain-bound representations and processes, as advocated most strongly by the embodied approach, as we saw representations (see Dawson, 2013; Richardson and Chemero, 2014). The disputes concerning the “non-representational” approach, however, are still ongoing and are not yet conclusive. An interesting stance is the notion of *minimal representationalism* in this regard, which posits that action-oriented representations can function as internal states that describe aspects of the world and that prescribe possible actions (Clark, 1997; Engel, 2010).

Two major questions keep popping up, however: what is the role of direct conscious control of movements? And should we rely on internal representations of previous learned movements? A possible answer lies in the learned associations between action and effect (Elsner and Hommel, 2001; Hommel et al., 2001), which, in the case of musicians, goes over years and thousands of hours of deliberate practice. Learning to play an instrument, then, might be considered as an extreme case of *action-effect coupling* (A-E coupling) in which certain actions are carried out to produce certain sounds (Drost et al., 2005). Such couplings can be used for the voluntary control of actions with an initial stage in which associations between action codes and effects are established, and a second stage in which these associations are used for controlled behavior by activating involuntarily the associated action representation by imagining the desired effects. Movements, in that view, are selected by anticipating or activating the codes of their consequences (Elsner and Hommel, 2001), as stated also in the “ideomotor” theory of action control (Hommel et al., 2001; Prinz and Chater, 2005). According to this model, musicians must merely imagine a musical sequence to automatically activate the associate sequence of appropriate actions without need for direct conscious control of the actions themselves. Evidence for such activation comes from so-called induction errors studies where participants erroneously play a perceived musical interval rather than the interval they were required to play. It means that the potential action effect drives their motor routine with faster reactions than those which are consciously controlled (Drost et al., 2005). Such coupling of action representations and sensory action effects pertain to almost all actions, with a decrease in executive actions control as a result of practice. It explains to a major extent the movement aptitude in skilled behavior like instrument playing. Motor skill learning, further, involves the planning and execution of movements with increasing spatial and temporal accuracy of movements as the outcome of practice. Its neural basis, however, is still somewhat elusive. Many brain areas are involved in the process, with multiple links to functional systems such as perception, attention, and memory.

Additionally, a significant aspect of learning hinges on the neural separability of various cognitive components within motor control. This is exemplified in the *dual mode principle*, which claims two different forms of representation which allow motor acts to be executed either in a conscious, effortful mode or in an unconscious, automatic mode, relying on anatomically distinct parts of the brain (Willingham, 1998). The case of music performance is extremely instructive here. Expert performance requires the initiating, controlling, and adjusting of difficult action sequences. It should be noted, however, that self-conscious thought can disrupt well-practiced action. Grooved action sequences of the body, therefore, may be entrusted to the habitual routines of kinesthetic memory. Open-ended, flexible performance, however, is context-sensitive and responsive to subtle changes in a situation. Memory and movement and thought and action should be brought together instead of competing each other (Sutton et al., 2011).

### 3.2 Sensorimotor interactions

Viewing music performance as a form of knowledge construction involves an approach to musical sense-making that emphasizes the actual experience of the music as it is performed. When approached from a disembodied perspective, this view may seem to place significant emphasis on the sensory input and its processing – as in classical cognitivist scenarios. Care should be taken, however, not to rely too restrictively on the “linear” input–output model of information processing. A more promising approach, known as the “circular model,” discards the conventional input/output divide and introduces a recursive system. This system relies on the mutual specification of exteroception, interoception and proprioception. It shifts away from the traditional one-way setup that begins with an input and ends with an output, focusing instead on a circular relationship where each parameter interacts and influences each other in a mutually defining manner (Schiavio et al., 2022d). It is an approach that echoes somewhat the distinction between “exafference” and “reafference,” with the former referring to external stimuli and the latter to self-generated stimuli, which originate, respectively, from outside of the body and from within (see Bays et al., 2006; Voss et al., 2006; Cullen, 2012; Reichenbach and Diedrichsen, 2015; Jékely et al., 2021). However, this distinction may appear somewhat artificial: when external sounds penetrate the body and resonate within its deeper tissues, they can also assume an interoceptive dimension (for a detailed discussion, refer to Reybrouck, 2023b). This dynamic interplay forms a loop that transcends rigid adherence to the concepts of inputs and outputs.

At this point, a distinction should be made between solo performing and performing together with others, but in each case, multiple fascinating couplings that go beyond the input/output dichotomy can be observed. The most obvious ones are the *auditory-motor* and the *visual-motor coupling*, which can be subsumed under the umbrella perspective of synchronization and embodied interaction with the music. Examples of auditory-motor coupling are music-to-movement synchronizations such as dancing to the music, tapping the beat, singing along with heard music, playing together with other people, and even imagining the heard music at an internal level of ideomotor simulation. Examples of visual-motor coupling are score-to-movement synchronization or playing simultaneously with the movements of a conductor or other performers.

As to the “auditory-motor coupling,” it should be mentioned, first, that the auditory system is faster and more precise than the visual and tactile ones (Shelton and Kumar, 2010). It can prime and time muscle activation via reticulospinal pathways with richly distributed fiber connections to motor centers in the spinal cord, the brain stem, and even up to subcortical and cortical areas. Taken together, these can activate an auditory-motor circuit for entrainment that is linked to the time and frequency dynamics of the sound stimuli with potential interesting clinical applications in the field of motor function rehabilitation (see Thaut et al., 2015 for an overview). Yet, despite their primary importance in music performance, these findings did not yet receive much attention in motor control theory and motor rehabilitation, most of which has been directed rather at visual and proprioceptive stimulation. Expert musicians, further, tend to associate the sounds from their instrument with the movements that produce them and vice versa. This is a *sound-action association* which is the result of learning the contingencies between sounds and movements over longer periods of training (Lahav et al., 2007; Chen et al., 2012). This may facilitate or prime the corresponding actions, even when they are not relevant to the task, and the performance of movements can even alter the perception of the sounds (Drost et al., 2005; Brown et al., 2015). The auditory-motor interactions, further, rely on general-purpose pathways with two paths, namely the above-mentioned dorsal and ventral route (Hickok and Poeppel, 2007; Rauschecker and Scott, 2009). The ventral component seems to be important for the processing of melodic contour and intervals, auditory pattern recognition and object identification, while the dorsal component is the most relevant for the sensorimotor aspect of music performance. It engages dorsal parietal and premotor circuits when they transform sound patterns into motor patterns (Lee et al., 2011; Brown and Palmer, 2013; Klein and Zatorre, 2014).

The “visual-motor coupling,” on the other hand, has major applications in sight-reading. Many questions, however, are still open here. One of them is related to musical imagery and notational audiation, assuming that musicians can hear an inner voice—analogous to subvocalization, inner voice, or inner speech (see Aleman and Wout, 2004 for a critical comparison), while reading musical notation. Studies on mental representation of music notation have revealed that highly trained musicians relay on music imagery as much as on the actual external sounds they produce (Hubbard and Stoeckig, 1992), relying on the specific skill of hearing the music they read before physically performing it. It is a claim that revitalizes Gordon’s notion of *audiation*, namely the internal analog of aural perception (Gordon, 1997). His claims have been corroborated by evidence that brain areas that process auditory information are recruited also when this information is internally generated (Halpern, 2001). It has been suggested, therefore, that notational audiation elicits kinesthetic-like covert phonatory processes analog to silent singing (Brodsky et al., 2008) and musicians who display notational audiation skill largely rely on silent reading through the piece before playing (Brodsky et al., 2008).

The findings echo somewhat the distinction made by Smith between the inner ear and the inner voice, with notational audiation being related to the inner voice rather than to the inner ear (Smith et al., 1992; Brodsky et al., 2003). It highlights the fact that many cognitive abilities depend on both perceptual and motor processes. Silent music reading, in that view, typically illustrates the cross-modal encoding of a unisensory input. This cross-modal encoding, however, is not obligatory, but is the result of a strategic type of sensory interaction that develops after explicit learning, effortful processing, and considerable practice (Brodsky et al., 2008). The underlying mechanism involves intersensory and sensorimotor—also movements have their sensory modalities, such as proprioception and the kinesthetic sense—translation processes that turn the symbols of the notes into adequate responses. Such coupling mostly proceeds in two stages of learning: an early stage that establishes associations between action and effect codes, and a stage in which the associated actions take place by simply imaging the desired effect (Drost et al., 2005). This aligns with the above-mentioned “ideomotor” claim that trained expert musicians can activate a sequence of actions by merely imagining or anticipating a music sequence without need of direct conscious control of the movements. It can be questioned, however, whether this holds equally strong for both music performance and music reading (Brodsky et al., 2008). An interesting question, further, is the role of visual–auditory coupling, which seems to mediate between the visual input and motor output. There is the frequently observed phenomenon of subvocal speech while reading, and expert musicians report hearing the music when they read a score (Brodsky et al., 2003). A distinction must be made, however, between cross-modal encoding which appears to be quasi-automatic—as in the case of synesthesia—and obligatory and auditory recoding of text and music, which reflects an effortful processing strategy (Guttman et al., 2005).

### 3.3 Representation and imagery

The concept of representation has recently been challenged in cognitive science with discussions between so-called representational-computational as against non-representational, dynamic-enactive approaches. We briefly engage with this topic by looking at the concept of *embodied dynamicism* (see Reybrouck, 2021 for a broader overview). This is an approach that takes a critical stance toward computationalism, which heralds a disembodied and abstract model of cognition without any relationship to the living body of an organism and its environment. Rather than conceiving of the mind and the body as separate and independent of each other, with the outside world being mirrored by representations inside the head, it focuses on the dynamics of cognitive processes that emerge from continuous sensorimotor interactions which involve the brain, the body, and the environment. The mind, in this view, is an embodied dynamical system that involves brain, body and world rather than an encapsulated system in the head (Thompson, 2007, p. 11). Cognition, then, is only possible when a relationship between a brain–body system and the world is established.

The embodied dynamicists approach must be positioned in the context of *enactivism*. Being defined by its founders in terms of an organism’s sensorimotor capacities, which are embedded in and engaged with the wider context of the biological, psychological, and cultural varieties (Varela et al., 1991, p. 172), it was conceived as an antidote to those disembodied approaches to mind that take representation and computational processes within the mind as their central notion (see Hutto and Myin, 2013 for an overview). Central in this radical embodied approach is the idea that organisms “enact” or “bring forth” their worlds, and that enaction enables a world to “show up” for individuals (Maturana, 1987). Reality, in that view, is not pregiven, but must be constructed, setting up a dynamic system in which the meaning of the world amounts to the consequences of the organism’s actions for its sensory inputs. This is the core characteristic of enaction (Stewart, 2010; Stewart et al., 2010). A related idea has been embraced also by the *pragmatic turn* in cognitive science with a shift from the traditional representation-cantered framework toward a paradigm that understands cognition as skillful activity that involves ongoing interactions with the world (Varela et al., 1991; O’Regan and Noë, 2001; Noë, 2004; Kurthen, 2007; O’Regan, 2011). The case of music performance is interesting in this regard. There is the sensorial aspect of capturing self-produced sounds which proceeds in real time, but this pure perception is mostly accompanied by cognitive operations that work also outside of the time of real sounding. There is, as such, an interplay between physical interactions with the sound-producing devices (the instrument) and the actual modulation of the sounds and virtual or epistemic interactions, such as comparing, recalling, anticipating, transforming, and other mental operations which are performed at the level of imagery or symbolic computations, and which are not constrained by the inexorable character of the unidirectional unfolding of time.

Traditional models of representation have been inspired rather narrowly by the computer metaphor to describe how perception, cognition, and action can occur. The term “motor program,” which was introduced to describe how the brain produces consistent and reliable movement outputs,” is perhaps most typical of this analogy (Keele, 1968). It should be seen in the context of the Schmidt’s schema theory of *discrete motor skill learning* (Schmidt, 1975), which combines aspects of open and closed loop control. Schemas, in this view, are sets of rules that link the response of the execution of a movement to feedback that is received during and after performance. A generalized motor program, in Schmidt’s view, is an abstract representation that contains the general characteristics for a given class of movements. It states that variable, rather than specific, practicing conditions are necessary to acquire a robust schema to generate functional movements for a particular class of actions under a variety of environmental conditions. Such generalized motor programs counter the idea that each separate motor program should be stored, in favor of representing a whole class of actions. Examples are stepping, walking, running, skipping, and gambling, which together form the class of locomotion. Learners, then, must learn to set key specifications or parameters such as speed, duration, and force of movement, which, as the result of practice, should rely less on feedback and develop into open-loop control via parameter regulation with a minimum of cognitive burden. This may lead to the formation of schemas, which are the result of the information that is stored during the acquisition of skills, and which embrace the initial conditions, the response specifications, the sensory consequences, and the response outcome. Linking these together, then, entails the construction of *recall* and *recognition schemas*: the former are used to start movement production; the latter to evaluate the accuracy of the selected movements (see also Davids et al., 2008).

Since Schmidt’s initial assertions, there has been considerable theorizing and empirical research. A crucial aspect is the perceptual representation of the action effect, serving as a retrieval cue for selecting the most suitable or effective action aligned with the action goal (Elsner and Hommel, 2001). According to this perspective, actions are encoded by anticipated effects, suggesting that movement patterns and their perceptual outcomes are automatically integrated and stored as learned outcomes. Learning is thus conceptualized as the construction of internal models of the world and movements to enhance actions and interactions with the environment (Van Orden et al., 2003). Nonetheless, early theories encountered challenges related to specificity, storage capacity, and computational complexity. Recent theories of representation, however, can take advantage of the developments in fields such as computational network theory, neuroscience, artificial intelligence, and robotics to explain how learners can acquire adaptive and reliable movements. There is, in other words, an increased understanding of the role of the central nervous system in modeling the representational control of behavior (Davids et al., 2008), and neuroscience seems to have found a solution to measuring internal phenomena in the brain by relying on functional imaging techniques, which make it possible to measure them directly rather than by inference (Brodsky et al., 2008). It is still a major issue, however, to establish “what” to measure as there is no evidence that placing subjects in a scanner does guarantee that they perform the intended mental activities. It makes sense, therefore, to combine behavioral paradigms of measurement from experimental psychology and psychoacoustics with neuroimaging techniques to uncover the underlying mechanisms of the internal phenomena.

The overall findings present a complex landscape, particularly concerning the relationship between representation and imagery. While extensive research has delved into the visual modality, recent studies have increasingly explored auditory imagination, expanding our understanding (see Godøy and Jørgenen, 2001; Küssner et al., 2023 for a comprehensive overview). Recent discoveries appear to support earlier influential works that underscore the significant role of the kinesthetic sense in generating imagery (Seashore, 1938) and the interconnectedness between aural and oral channels (Reisberg, 1992). Moreover, contemporary neuroscience studies have revealed the activation of motor circuits in the brain during music imagery, particularly during covert mental rehearsal (Halpern, 2001; Langheim et al., 2002; Baumann et al., 2007). This highlights the auditory-motor connections that enable the experience of music even when it is not physically present.

The role of inner speech or subvocalization—the experience of hearing an inner voice without vocal output or environmental input—has been important in this regard, with findings that its auditory quality is not necessarily auditory in origin, but that it is linked to the phonological system, which clearly shows that there is no single seat for auditory-based imagery (MacKay, 1992; Brodsky et al., 2003). Musical images, moreover, are generated in real time and encode precise information about tempo and pitch and melodic and harmonic relationships. As such, they have sensory qualities that are similar to the experience of perceiving (Janata, 2001a,b), but this seems to be the outstanding hallmark of a trained musical mind rather than musically naïve individuals (Aleman et al., 2000). The skill of *notational audiation* is quite interesting in this regard. It has been defined by Brodsky as “the engagement of kinesthetic-like covert excitation of the vocal folds with concurrently cued motor imagery” (Brodsky et al., 2008, p. 443). Despite its elusive character, there is now a beginning of conclusive agreement on the nature and developments of its underlying mechanisms, with interesting findings about the respective role of auditory, phonatory or manual-motor resources. It has been found also that movement representations of music performance facilitate performance with two major findings: there is a reliance on kinesthetic phonatory and manual processing during subvocalization when reading music notation, and the mental representation of the notation entails a dual-route stratagem, namely the aural-oral subvocalization (internal kinesthetic image of the inner voice) and aural-motor impressions (internal kinesthetic image of music performance; Brodsky et al., 2008). The transition from imagery to actual performance, further, entails separate representations in motor control, namely a strategic process, perceptual-motor integration and sequencing, and a dynamic process. The strategic process is related to goal selection and perception and uses representation of *allocentric space* with objects being located and coded relative to one another but outside of the body; the perceptual-motor and sequencing process supports actual motor behavior and uses *egocentric space* where the object’s location is coded relative to some part of the body; the dynamic process, finally uses representations of muscle activity (Bridgeman, 1991; Paillard, 1991; Jeannerod, 1994 and Willingham, 1998 for an overview).

## 4 Performance as cognition?

There is a long history of perceptual theories of knowledge. A pivotal moment in this history is represented by Barsalou’s theory of perceptual symbols (Barsalou, 1999). While heavily rooted in perception, this theory extends into the realm of knowledge, asserting that neuronal systems in the brain’s sensory-motor regions capture qualitative and functional information about perceived events in both the environment and the body. In essence, perceptual symbols are essentially records of the neural activation that occurs during perception, serving as a foundational principle in modern perceptual theory. There is no place to go into detail here. It may suffice to highlight some basic tenets. One of them is the interpretative process that is inherent in cognition, in the sense that cognitive representations are not holistic bit-mapped recordings (Pylyshyn, 1973; Hochberg, 1998). They are rather interpretations of experience. It has been argued, in this regard, that abstractions—mostly in the form of propositions—underlie these interpretive processes, with concepts functioning as a summary representation to support later interpretations of experience. Such abstractions, however, are difficult to specify with notoriously three problems to be solved: identifiability (what information should be included?), justification (how to justify the inclusion of particular information?), and rigidity (robustness against exceptions) of the used categories (Barsalou, 2003). They show the need of better methodologies to uncover possible abstractions. One possibility is to argue for an infinite number of abstractions instead of one single abstraction to represent a category. These should then be constructed dynamically to represent a category temporarily.

The framework of perceptual symbol systems meets these conditions (Barsalou, 1999, 2003). Starting from an assumed *convergence zone architecture,* it states that once conjunctive neurons in a convergence zone in the brain capture a pattern of activation in some feature area, they are able to reinstate that pattern in the absence of sensory stimulation. Or put in other words: while perceiving an object, conjunctive neurons re-enact the sensory-motor and introspective states that were active during the original processing of that object. The re-enactment is modality-specific and is never complete, but at least some semblance to the original state is partially reinstated. Subsets of such perceptual states can be extracted, moreover, and stored permanently in long-term memory to function symbolically on later retrievals. As such, collections of perceptual symbols may develop, standing for referents in the world, thus opening the possibility of symbol manipulation. There are, in this regard, two attentional assumptions that are quite axiomatic in cognitive psychology: selective attention isolates information in perception and it stores this information in long-term memory. A major assumption of perceptual symbols theory is that a common representational system underlies perception and cognition. Perceptual symbols are modal (and even multimodal) and analogical, which means that they are represented in the same systems as the perceptual states that produced them. This sets them apart from computational theories of representations that use amodal symbols and that use a representation language that is inherently non-perceptual. It can be asked, finally, to what extent perceptual symbols may be related to motor learning and control. Can we conceive merely of “perceptual symbols” or should we extend the concept to “perceptual-motor symbols” which can be used also in the execution of new and previously established motor acts? The question opens up perspectives somewhat related to the “common-coding theory,” which states that actions are coded in terms of their perceptual effects (Hommel et al., 2001), and the “theory of internal models,” which assumes that forward models can generate predictions of the sensory consequences of actions, which can be compared with the actual sensory input (Wolpert and Kawato, 1998). The theories claim effects of both perception on action, and of action on perception.

Translated to the realm of music, this means that performing is not only action-oriented. Equally important is the whole machinery of multiple sensory feedback loops, which act as tools for an ongoing process of knowledge construction, both short-term and long-term. High-level performing involves listening. It provides first-hand information of the accuracy and quality of the produced sounds, relying heavily on continuous and focal attention. Such heightened state of alertness increases the vitality of the musical experience by celebrating the sensorimotor couplings that turn the open loops into closed loops, or put differently: there is a shift in direction from “centrifugal” to “centripetal” processing of the sounds in the sense that the sounding effects of the motor output function as a new input to the perceptual system. It is a typical example of the principle of circularity with a continuous transition from output to input, and input that generates new or modified output. Performance, in this view, overlaps with “learning,” conceived of as knowledge acquisition. It echoes somewhat the claims by James in his epistemological doctrine of radical empiricism, in which he stressed the role of knowledge-by-acquaintance, as the kind of knowledge we have of a thing by its presentation to the senses. Conceptual knowledge may be self-sufficing, but the significance of a concept consists always in its relation to perceptual particulars. Or as he puts is: “We extend our view when we insert our percepts into our conceptual map (…) but the map remains superficial through the abstractness, and false through the discreteness of its elements. […] Conceptual knowledge is forever inadequate to the fulness of the reality to be known.” (James, 1976, p. 327).

James’ insights are still inspiring. They argue against the disembodied view on knowledge which has dominated cognitive science for decades by stressing the hegemony of conceptual over perceptual knowledge. Both kinds of knowledge do not exclude each other, however. They are complementary in the sense that they conflate the inner/outer dichotomy. Performance, then, is a multifaceted process: it involves, among others, score reading and decoding the printed symbols, enacting the printed music in imagery, transforming the visual input into motor patterns, reproducing of stored procedural knowledge in case of playing from memory, adjusting the performed actions with respect to accuracy and expressivity by means of continuous and immediate sensory feedback, trying out newly create musical configurations in case of improvising, all this happing in real time. Many of these processes function partially unconsciously as during preconscious processing and automatized skills, with less cognitive efforts as a result of prolonged training. Yet relying merely on automatized skills can be dangerous as there is always the risk of memory failure or lapse in attention in a live performance. Trained musicians, therefore, may invest in the establishment of *performance cues* to which they can deliberately attend to during performance without disrupting the automaticity of highly practiced motor sequences (Chaffin et al., 2002, 2009). Such performance cues are landmarks in the mental map of the piece. They are monitored by musicians to ensure that critical aspects of the performance go as planned and are created during practice by repeatedly attending to particular features of the music so that they come to mind automatically while playing. They act as a safety net against disruptions in performance, in case that the normal serial chain of cues is broken.

Crucial in this is the tension between the conscious and unconscious mode of processing with a typical example in the case of learning. The conscious mode is typically engaged in the performance of an unfamiliar task, as the unconscious mode would lead to inaccuracies in performing. New motor tasks, as a rule, are attention-demanding, but these demands decrease with practice. This is the basic hallmark of automaticity. After gaining experience with a task, the unconscious mode may take over with sufficient accuracy so that the conscious mode must not be invoked. But even a well-practiced skill can be executed in the conscious, attending-demanding way of a novel skill or when the task becomes extremely difficult. It is possible, therefore, to engage the conscious mode at any time (Willingham, 1998).

It can be asked, finally what is the role of *expressive behavior* in this regard. The ability to communicate expressive contents in musical performance is a highly acquired skill (Lehmann and Davidson, 2002). It seems to be a quality that is added to skilled execution, relying heavily on the monitoring of the ongoing performance to ensure that the desired expressive quality is conveyed appropriately. Such monitoring is provided by various types of sensations that result from visual, tactile, proprioceptive, and vestibular feedback during performance in addition to the primary auditory feedback (Gabrielsson, 2003). It is important, however, that this feedback is monitored consciously, so as to become an essential constituent of performance cues. They include expressive cues, besides interpretive cues, structural cues, and basic cues with the last two referring to formal structure of the music and the critical details of technique that must be executed as planned for the performance to unfold as intended. Many of these become automatic as the result of practice, but only when they are singled out for continued attentions, they become performance cues (Chaffin et al., 2009).

## 5 Conclusion and perspectives

In this paper we have expanded on the topic of motor learning and control from an embodied perspective, with a special focus on music performance. We have raised the question to what extent playing a musical instrument can contribute to one’s construction of knowledge. We have advocated a centripetal approach to music performance, contrasting the conventional centrifugal perspective. In this context, the sounds generated during the performance not only emanate from the body (centrifugal), but they also circle back to it (centripetal). By this view, playing music is not only output-oriented; it rather involves a dynamical integration of external and internal factors. We selectively reviewed some older theoretical frameworks on motor learning and control as well as on expert behavior with the aim to confront some of the older seminal ideas with more recent theories on sensorimotor coupling and integration. As opposed to many contributions in the field of expert behavior, we also have not focused primarily on the “acquired state” of skill acquisition, but rather on the “acquisition stage”.

Focusing on such a genetic approach rather than on the description of already established and accomplished skilled behavior is quite instructive to find out how performers come to grips with the music both as it sounds and how it is constructed. The path from initial sight-reading or playing by ear to the final execution, in fact, embraces multiple mental and motor operations, such as exploration, attention, memory, perception, motor planning sensorimotor integration, automatization, and expressive behavior. As such, offering a framework that recognizes the centripetal nature of music performance may be of pivotal importance also for practical areas such as education: examining how the sounds produced during performance not only stem from bodily action but also cyclically revert to it may unveil fresh insights into the acquisition and development of skills, in turn providing new ways of thinking about pedagogical practice. This shift in analytical focus extends beyond one’s bodily movements to a broader spatio-temporal domain encompassing the body, its space of action, and the sonic environment that evolves during musical performance. In other words, the emphasis in music teaching and learning should not solely be on how musical sounds are generated but also on how they cyclically shape performative behavior, fostering new synergies that integrate open and closed loops, which may be both one-offs and repeated routines. Theoretically, the tensions between these two opens up interesting perspectives with respect to the challenged concept of representation. We are inclined to adhere to Barsalou’s conception of *perceptual symbols* as a starting point in this regard, but also the tension between *computationalists* and *dynamicists* is quite enlightening. To quote Thompson: “whereas computationalists focus primarily on discrete states and treat change as what happens when a system shifts from one discrete state to another, dynamicists focus on how a system changes state continuously in time.” (Thompson, 2007, p. 43). Cognition, on this view, is seen as the flow of complex temporal structures that mutually and simultaneously influence each other. Our literature review has revealed that, in a similar manner, music performance might be better understood as an ongoing cycle that enables performers to acquire knowledge in the process. This shifts the common perspective of performers simply as interpreters and sharers of their own knowledge. While these traditional roles may remain valid to certain extents, we emphasize the importance of recognizing the process of *knowledge acquisition* as a significant part of music performance. The claims, however, are still waiting for more broader theorizing and additional empirical support. As such, this viewpoint opens up challenging perspectives for future research.

## Author contributions

MR: Writing – original draft, Writing – review & editing. AS: Writing – review & editing.

## Footnotes

^1^This idea is inspired by the circular dynamics at the foundation of human cognition, as viewed from an embodied perspective (Fuchs, 2020). As such, it should be noted that our proposal does not lay claim to inventing something new; after all, it draws on older concepts that may appear novel when revisited or when applied to a specific domain.

^2^We have been inspired to some extent by Barsalou’s claim about the assessment of scientific theories, which depend not only on the ability to fit data, but which must be “evaluated on falsifiability, parsimony, the ability to produce provocative hypotheses that push a science forward, the existence of direct evidence for their constructs, freedom from conceptual problems in their apparatus, and integrability with theory in neighboring fields” (Barsalou, 1999, p. 580).
